# Role of promoting inflammation of Krüppel-like factor 6 in acute kidney injury

**DOI:** 10.1080/0886022X.2020.1793353

**Published:** 2020-07-23

**Authors:** Dan Li, Xiaoqiang Liu, Chenyu Li, Yue Zhang, Chen Guan, Junyan Huang, Yan Xu

**Affiliations:** a Department of Nephrology, The Affiliated Hospital of Qingdao University, Qingdao, China; b Department of Nephrology, Qingdao Central Hospital, Qingdao, China; c Reproductive Medicine Center, Qingdao Women and Children's Hospital, Qingdao, China; d Division of Nephrology, Medizinische Klinik und Poliklinik IV, Klinikum der Universität, Munich, Germany

**Keywords:** Krüppel-like factor 6, acute kidney injury, ischemia–reperfusion injury, proximal tubule epithelial cells, inflammation

## Abstract

**Background:**

Krüppel-like factor 6 (KLF6) is a transcription factor that participate in various pathophysiological processes, but its contribution in ischemia acute kidney injury (AKI) is lacking so far. The study aimed to investigate the expression and the role of KLF6 in kidney ischemia–reperfusion (IR) injury.

**Method:**

Microarray data were collected from GSE58438 and GSE52004. The rat IR model was established to evaluate the mRNA and protein expression of KLF6 and inflammatory cytokines in serum and kidney tissues. SiRNA-KLF6 was transfected with HK-2 cells, and then a cell-based hypoxia-reoxygenation (HR) model was established.

**Results:**

Bioinformatics showed KLF6 mRNA in kidney tissue is up-regulated in 3 h after IR in rat kidney, which involved in cell activation, leukocyte activation, and response to hydrogen peroxide after IR. The rat IR model results showed that KLF6 expression was peaking at 6 h, and the expression of pro-inflammatory cytokines MCP-1 and TNF-α was increased both in serum and kidney tissues, while anti-inflammatory cytokine IL-10 was decreased after IR. Furthermore, *in vitro* results showed that KLF6 knock-down reduced the pro-inflammatory cytokines expression.

**Conclusion:**

These results suggest that (1) KLF6 might be a novel biomarker for early diagnosis of AKI and (2) KLF6 may play a role in promoting inflammation in AKI.

## 1. Introduction

Acute kidney injury (AKI) is always associated with high morbidity, mortality, and increased costs of treatment in both pediatric and adult patients [1]. The worldwide annual incidence of AKI is approximately 2100/million [2], and the number of AKI-caused deaths is approximately 2 million [3], of which 20–25% are inpatients. In particular, critical AKI caused more than 50% mortality rate during dialysis [4]. Among AKI risk factors, ischemia–reperfusion (IR) is the leading as well as underlying multi-factorial pathophysiological process [5] which affecting both the renal tubular epithelium and renal microvasculature [6,7]. Many researches also have provided strong evidence that oxidative stress and inflammation are major contributors to the pathogenesis of ischemic AKI [8–12]. In early phase of IR, inflammation is alloantigen independent and is characterized by activation of not only classical immune cells but also resident renal cells, such as endothelial cells and tubular epithelial cells (TECs) that are extremely sensitive to oxidative stress [13]. The inflammatory factors and adhesion molecule produced by TECs cause a large number of inflammatory cells to migrate and infiltrate, further aggravating renal damage, and subsequently, inflammation amplification [14–16]. Complete recovery of kidney function enjoy a better outcome, but partial function recovery may occur due to significant irreversible nephron loss caused by inflammatory, the possibility of a progression to chronic kidney disease (CKD).

As a member of the Krüppel-like factors (KLF) family, KLF6 contributes to cell proliferation, differentiation, apoptosis, and autophagy, which also has an essential role in tumor suppressor that is inactivated or downregulated in different cancers including prostate, colon, and hepatocellular carcinomas [17–19]. KLF6-related researches on the kidney revealed that KLF6-dependent regulation of the cytochrome c oxidase assembly gene which is critical for maintaining mitochondrial function and preventing podocyte apoptosis [20]. KLF6 also plays a permissive role in TGF-ß1-induced epithelial-mesenchymal transition (EMT) in proximal tubule cells *in vivo* models of diabetic nephropathy [21]. In 2005, Tarabishi et al. have demonstrated the rapid and dramatic up-regulation of both KLF6 and TGF-ß1 in ischemia AKI [22].

Studies found that KLF6 can promote pro-inflammatory gene expression while restraining anti-inflammatory gene expression in macrophages. Therefore, KLF6 toggle controlling macrophage speciation [23–25]. Similarly, research revealed that KLF6 is a co-activator of NF-κB p65 for transcription of selected downstream genes [26]. Moreover, KLF6 strongly associated with T-cell activation in psoriasis [27], and KLF6 knockout mice showed a protected effect to against chemically induced colitis, suggesting a central role of myeloid KLF6 in pro-inflammatory activation in the setting of chronic intestinal inflammation [24]. Nonetheless, there are no studies evaluating the role of KLF6 in renal inflammation following AKI.

In this study, we investigated (1) the expression of KLF6 and inflammation cytokines *in vivo* and *in vitro* model of IR injury, and (2) through knockdown of KLF6 to investigate the potential effect of it on inflammatory cytokines during IR.

## 2. Materials and methods

### 2.1. Bioinformatics analysis

We searched the public microarray database of GEO (http://www.ncbi.nlm.nih.gov/geo/) for AKI-related samples. Raw data of series GSE58438 [28] and GSE52004 [29] were downloaded from GEO database. GSE58438 was derived from total kidney mRNA that rat unilateral kidney I/R was produced. And GSE52004 was derived from four different types of translating ribosome affinity purification (TRAP) of mRNA populations from mice bilateral kidney I/R. The *oligo* package [30] was applied to assess the microarray quality. Robust multi-array averaging method [31] was used to preprocess the raw data after that. Probes were annotated using the latest official annotations file. Linear Models for Microarray Analysis [32] was applied to obtain differentially expressed genes (DEGs). For comparison of KLF6 fold change between AKI group and control group, moderated *t*-test [32] was used. False discovery rate (FDR) was calculated using the Benjamini–Hochberg method. Fold changes >2 or <0.5 with FDR < 0.05 were determined as the threshold to select DEG. All bioinformatics analysis was processed and analyzed by R software (v.3.4.1).

Gene set enrichment analysis (GSEA, http://www.broad.mit.edu/gsea) is a computational algorithm that inquiries a set of genes function and determines whether genes are statistically significant between two biological states [33]. The 3 h after I/R microarray data sets were used in which GSEA was performed to investigate KLF6 relative functions in AKI. Gene Ontology genes sets consisting of three sub-ontologies (Biological Process, Cellular Component, and Molecular Function) were used by annotated gene sets of GSEA, which were downloaded from the Broad Institute (http://software.broadinstitute.org/gsea/index.jsp). The *p* < 0.01 and the FDR < 0.01 were considered statistically significant for enrichment of gene sets.

### 2.2. IR animal model

A total of 40 male Sprague–Dawley rats aged 8 weeks and weighing 150–180 g were purchased from Vital River Experimental Animal Technology Company. Animals were housed in a light-controlled room with a 12-h light/dark cycle and were given free access to food and water throughout the study. The experiment was conducted in compliance with the Guidance Suggestions for the Care and Use of Laboratory Animals, formulated by the ministry of Science and Technology of the People’s Republic of China. Animals were randomly stratified into three groups with equal average initial body weight: (1) control group (*n* = 5); (2) Sham-operated group (*n* = 5); (3) I/R group (*n* = 25). Renal ischemia was induced by removing the right kidney and the left renal pedicle was cross-clamped for 45 min. After the renal clamps were removed, the blood flow to the kidney was reestablished with visual verification of blood return, rats were euthanized at 0, 3, 6, 12, 24h after the surgery (five rats at each time point). Sham-operated animals went through the same surgical procedure, including blunt dissection of the renal pedicle, but kidneys were not removed and renal clamps were not applied.

### 2.3. Bio-samples collection

Blood samples were collected from the left ventricle. The samples were centrifuged (4 °C, 3000 r/min for 10 min) to gather the serum. All serum samples were analyzed for biochemical parameters within 24 h after collection. Blood urea nitrogen (BUN) and serum creatinine (Scr) concentrations were measured using a Technicon RA-1000 autoanalyzer (Diamond Diagnostics, Holliston, MA) at the affiliated hospital of Qingdao University and used as indicators of renal function. Systemic perfusion with PBS through the left ventricle was carried out to wash out remnant blood. Kidneys were quickly removed, decapsulated, weighed, and evenly dissected into two parts from the coronal plane. One part was placed into 10% phosphate-buffered formalin and embedded in paraffin for immunohistochemistry and hematoxylin–eosin staining. The other part of kidney was quickly frozen and stored at −80 °C for further assay.

### 2.4. Hematoxylin–eosin (HE) staining

Paraffin-embedded sections (4 μm) were deparaffinized with xylene, dexylened in successive concentrations of ethanol, and stained with hematoxylin and eosin. Tissue sections (5 sections per kidney) were blindly labeled and randomly observed by two renal pathologists. A total of 10 fields per section were chosen randomly. Renal damage was graded based on the percentage of damaged tubules in the sample: 0 = no identifiable injury; 1 = mitosis and necrosis of individual cells; 2 = necrosis of all cells in adjacent proximal convoluted tubules, with survival of surrounding tubules; 3 = necrosis confined to the distal third of the proximal convoluted tubules, with a band of necrosis extending across the inner cortex; and 4 = necrosis affecting all three segments of the proximal convoluted tubule. Injury included inflammatory cell infiltration, dilation of renal tubules, and interstitial edema. A score of 1 or 2 represents mild injury, and a score of 3 or 4 represents moderate or severe injury, respectively.

### 2.5. Immunohistochemistry

The paraffin-embedded sections were incubated with primary antibody against KLF6 (Rabbit, polyclonal, Santa Cruz Biotechnology Inc., Santa Cruz, CA) overnight at 4 °C. For negative control, the sections were incubated with PBS alone. Specific labeling was detected with a biotin-conjugated goat anti-rabbit IgG and avidin–biotin peroxidase. Immunohistochemical staining for KLF6 was examined with light microscopy by two blinded reviewers. In each sample, five kidney sections were investigated, 10 fields per section were chosen randomly.

### 2.6. Enzyme-linked immunosorbent assay (ELISA)

The serum level of KLF6, ICAM-1, MCP-1, TNF-α, and IL-10 was tested by ELISA kits (R&D Systems, Minneapolis, MN) according to the manufacture’s instruction. All experiments were performed with triplicate samples and repeated three times. Renal tissues were homogenized and assayed with KLF6 and inflammatory cytokines (ICAM-1, MCP-1, TNF-α, and IL-10) by ELISA kits (R&D Systems, Minneapolis, MN) according to the manufacture’s instruction. A microplate reader was used to read the absorbance of each sample, and the results were calculated using the computer-generated linear curve-fit. The total protein concentration in homogenates was determined with a Braford protein assay kit (Bioteke Corporation, Beijing, China).

### 2.7. Cell culture and transfection

HK-2 cells were purchased from the Cell Bank of the Chinese Academy of Sciences (Shanghai, China). The cells were cultured in DMEM-F12 medium (12400024, Gibco, Carlsbad, CA) mixed with 10% fetal bovine serum (16141061, Gibco, Carlsbad, CA) and 100× penicillin–streptomycin solution (10 KU/m penicillin, 10 mg/ml streptomycin, P1410, Solarbio, China) and incubated in a 37 °C humidified incubator in an atmosphere of 5% CO_2_. For hypoxia treatment, cells were plated to 80% confluence, and the medium was replaced with glucose-free serum-free medium before HR treatment. HR group cells were exposed to 24 h of hypoxia (5% CO_2_, 1% O_2_, and 94% N_2_) followed by 6 h of reoxygenation for the experimental time period. Control cells were incubated under normoxic conditions without a medium change.

SiRNA-KLF6 and non-sense siRNA plasmid were purchased from Genechem (Shanghai, China). The siRNA plasmid was transfected with Lipofectamine 3000 (Invitrogen, Carlsbad, CA) according to the manufacturer’s protocol. HK-2 cells were cultured in DMEM-F12 supplemented with 10% heat-inactivated FBS in a six-well plate (3 × 10^5^cells/ml per well) and replaced by fresh DMEM-F12 medium with serum until the cells were 80% confluent. Lipofectamine 3000 reagent was diluted in serum-free DMEM-F12 medium (5 µl: 125 µl per well) and plasmid-P3000-serum-free medium complex (5 µg: 10 µl: 250 µl per well) was prepared. Two complex were mixed in a 1:1 ratio, incubated for 15 min at room temperature and then added to HK-2 cell culture dish evenly. The HK-2 cells ware incubated in a CO_2_ incubator at 37 °C for 72 h and then changed the medium for hypoxia/no hypoxia experiments.

### 2.8. Quantitative RT-PCR

The total RNA was extracted from renal tissues and cell culture supernatants and isolated with Trizol (Invitrogen, Carlsbad, CA). The first-strand cDNAs were synthesized from 2 g of total RNAs in a 20 mL reaction using Prime Script RT reagent kit (TaKaRa Biotechnology, Dalian, China). Quantitative PCR was performed in a Eppendorf Mastercycler ep realplex detection system (Eppendorf, Hamburg, Germany) by Faststart universal SYBR green master (Roche, Frankfurt, Germany) according to the manufacturer’s protocol. The relative mRNA levels were normalized to Actin and calculated as 2^−ΔΔCT^. All the primer sequences used for quantitative PCR are shown in Table 1.

**Table 1.** Primer sequences for quantitative PCR.

Primer	Forward	Reverse
KLF6	5′-CGGACGCACACAGGAGAAAA-3′	5′-CGGTGTGCTTTCGGAAGTG-3′
Mcp-1	5′-TCACCTGCTGCTACTCATTCACCA-3′	5′-AAAGGTGCTGAAGACCCTAGGGCA-3′
TNF-α	5′-ACCGTCAGCCGATTTGCTATCTCA-3′	5′-TGTAGGGCAATTACAGTCACGGCT-3′
IL-10	5′-GGGAAGACAATAACTGCACCA-3′	5′-TGTTTGAAAGAAAGTCTTCACCT-3′
ICAM-1	5′-GTGGTAGCAGCCGAGTCATAAT-3′	5′-CGTGGCTTGTGTGTTCGGTTTCAT-3′
Actin	5′-CCTGTGGCATCCATGAAACTAC-3′	5′-CCAGGGCAGTAATCTCCTTCTG-3′

### 2.9. Western blot

Renal tissues were homogenized in cold lysis RIPA buffer and centrifuged at 12,000 *g* for 30 min at 4 °C, and the supernatants (cytosol extract) were collected to evaluate contents of KLF6. The protein of cell culture supernatants was extracted using StrataClean Resin (Agilent Technologies, Palo Alto, CA) to evaluate contents of KLF6, ICAM-1, MCP-1 and TNF-α. Protein concentration was measured with a Braford protein assay kit (Bioteke Corporation, Beijing, China). Proteins were separated by polyacrylamide electrophoresis, transferred to a nitrocellulose membrane, and incubated overnight with monoclonal antibodies to KLF6, ICAM-1, MCP-1, TNF-α and Actin at 4 °C. Proteins were detected with a horseradish peroxidase conjugated secondary antibody 1:5000 in TBST containing 5% skim milk powder for 1 h at room temperature. The immunoreacted bands were quantified by digital densitometric imaging (Kodak 1D image analysis software).

## 3. Result

### 3.1. Klf6 was involved in cell activation, leukocyte activation, and response to hydrogen peroxide during AKI

**Figure 1.**
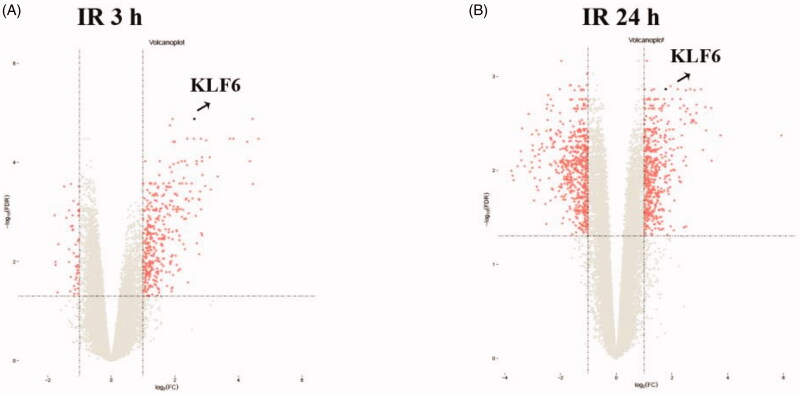
Volcano plot graph illustrating the differential abundant proteins in the quantitative analysis. The − log10 (*p* value) was plotted against the log2 (ratio IR/control). The black dots represented proteins level dysregulated in IR samples, Arrows indicated the KLF6 upregulation in IR samples.

**Figure 2.**
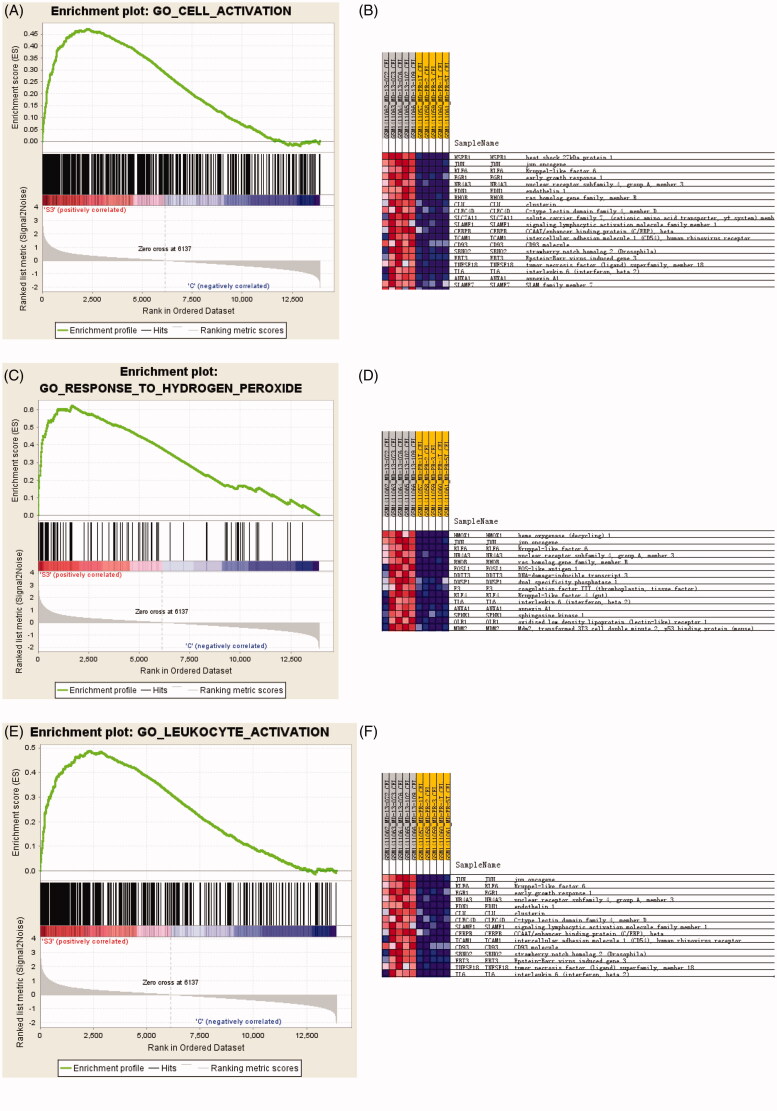
GSEA analysis of microarray data showing enrichment plots and heat maps for the gene sets of cell activation, leukocyte activation and response to hydrogen peroxide of the GSE58438 dataset. (A) Enrichment plot for the gene set of cell activation. (B) Heat map of the core enrichment genes of the gene set cell activation. (C) Enrichment plot for the gene set of hydrogen peroxide. (D) Heat map of the core enrichment genes of hydrogen peroxide. (E) Enrichment plot for the gene set of response to leukocyte activation (F) Heat map of the core enrichment genes of the gene set response to leukocyte activation. For enrichment plot, a positive ES indicates gene set enrichment at the top of the ranked list (the left part of the horizontal bar); a negative ES indicates gene set enrichment at the bottom of the ranked list (right part of the horizontal bar). The left part indicates positive correlation with IR and the right part indicates negative correlation with IR. Darker shades correspond to correlation values of greater magnitude.

Bioinformatics showed KLF6 mRNA in kidney tissue is up-regulated in 3 h and 24 h after IR compared with control group (Figure 1). GSEA revealed that KLF6 was involved in many critical pathways such as cell activation, leukocyte activation and response to hydrogen peroxide response to IR injury (Figure 2(A–F)). These findings suggested that KLF6 might be associated with AKI and participate in response to hydrogen peroxide functions and certain leukocyte activation.

### 3.2. Renal function and histology change in IR AKI

**Figure 3.**
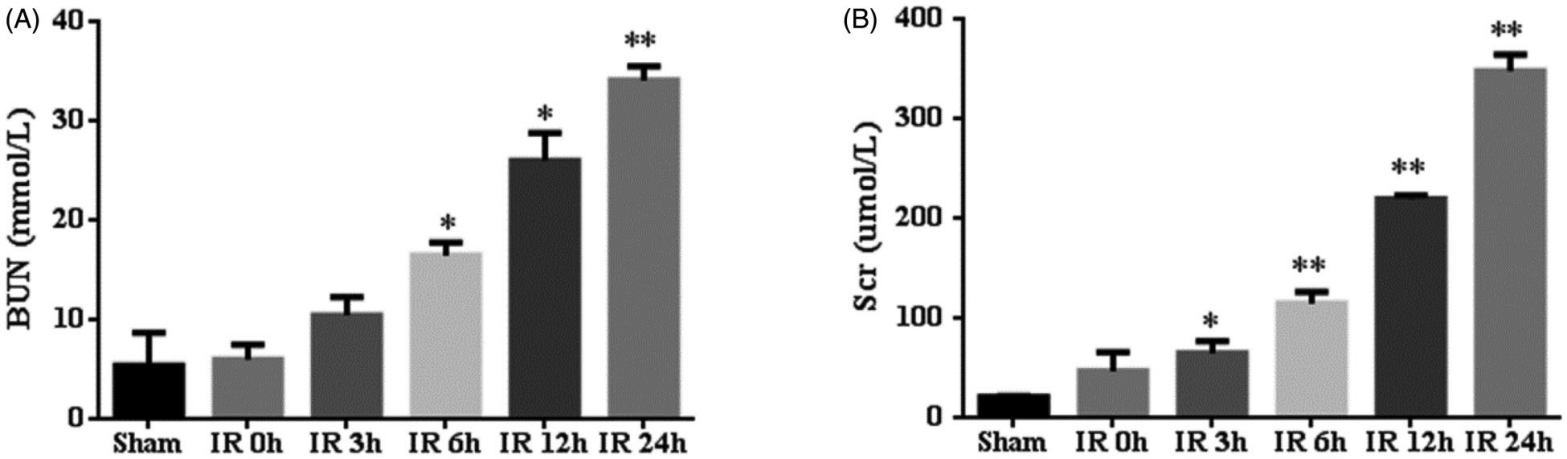
The renal function of mice with ischemia/reperfusion-induced acute kidney injury. Serum was collected at sham-operated group (*n* = 5) or renal ischemia/reperfusion injury (IR) (0 h, 3 h, 6 h, 12 h or 24 h, *n* = 5) in mice. Blood urea nitrogen (BUN) (A) and serum creatinine (Scr) (B) were measured. *p < 0.05 versus sham; **p < 0.01 versus sham.

**Figure 4.**
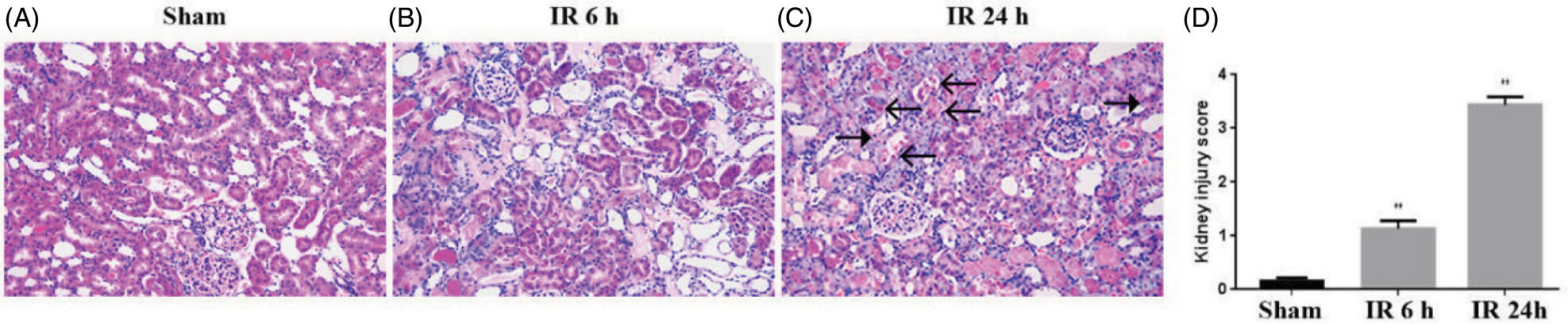
Pathological injury of renal tissues. The kidney histological changes were determined by HE staining (A: sham group, B: IR 6 h group, C: IR 24 h group) and (D) quantitative assessment of renal tubular injury. Arrows ←indicate macrophages, arrows ← indicate neutrophils. *p < 0.05 versus sham; **p < 0.01 versus sham.

To validate the bioinformatics results, we established IR AKI model, which sacrificed at 0 h, 3 h, 6 h, 12 h and 24 h after IR. Compared with sham operation, the IR group showed a significantly increased BUN and Scr levels, peaking at 24 h, suggesting IR AKI model successfully established (Figure 3(A,B)). Furthermore, HE staining showed that IR induced tubulointerstitial damage including focal areas of proximal tubular dilation and distal tubular casts, effacement and loss of proximal tubule brush border, and interstitial neutrophils and macrophages infiltration (Figure 4(A–C)). Moreover, the IR group presented mild pathological damage at 6 h (1.2 ± 0.2 versus 0.2 ± 0.1, *p* < 0.01)and most severe pathological damage at 24 h (3.4 ± 0.2 versus 0.2 ± 0.1, *p* < 0.01) than the sham group (Figure 4(D)), implying that the changes of renal function and histology damage are consistent.

### 3.3. Klf6 highly expressed in the kidney during IR

**Figure 5.**
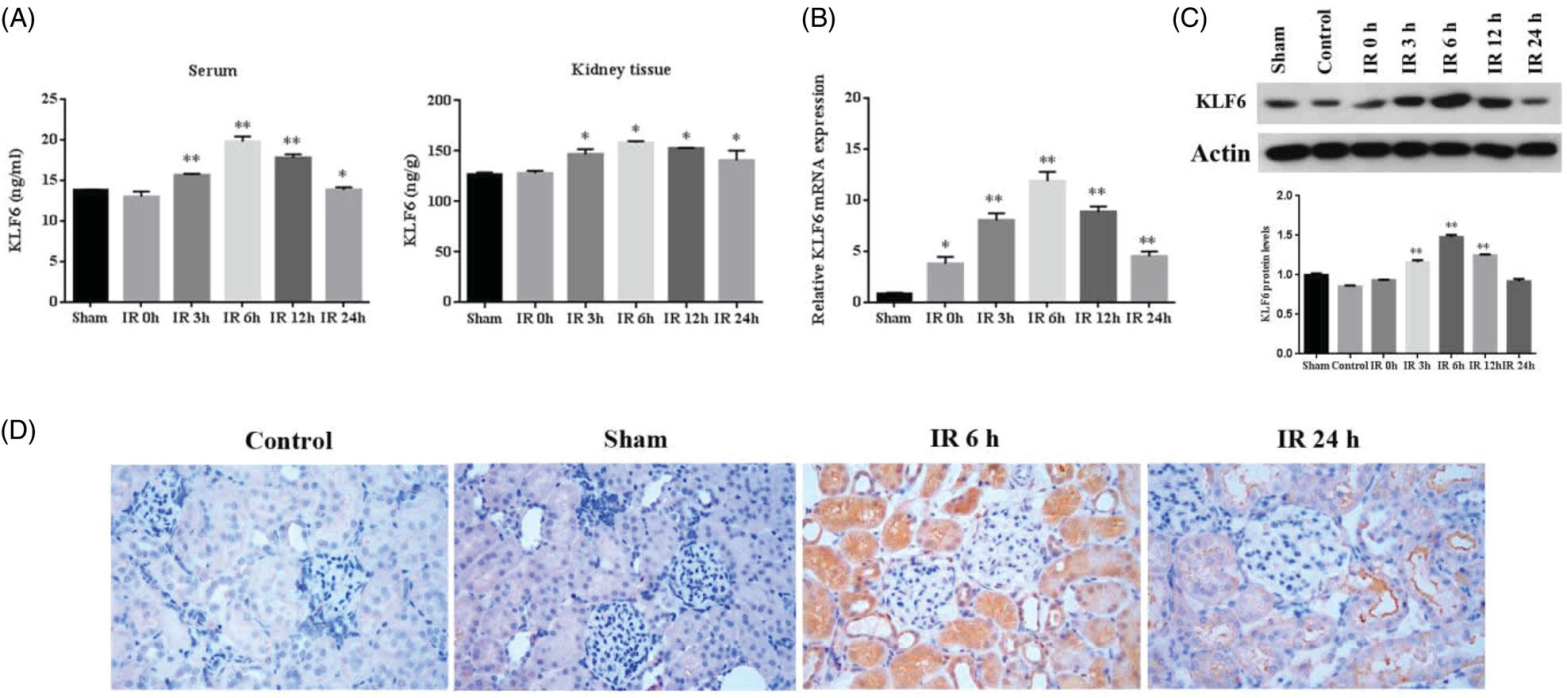
KLF6 expressed in the kidney during IR in the rat model. (A) Quantitative analysis of KLF6 released in the serum and kidney tissue using ELISA. (B) The expression of KLF6 was measured by RT-PCR, and the ratio of KLF6/Actin was normalized to the sham group. (C) The expression of KLF6 was measured by Western blot, and the ratio of KLF6/Actin was normalized to the sham group. (D) Representative photomicrographs of kidney sections Immunohistochemical staining for KLF6 in mice after sham or IR treatments . *p < 0.05 versus sham; **p < 0.01 versus sham.

To determine effect of KLF6 response to IR injury, we measured KLF6 mRNA and protein levels in kidney tissue and serum. First, we found that serum KLF6 protein level was highly expressed at 3 h and peaked at 6 h after IR, which was earlier than the BUN and Scr. Second, the expression of klf6 protein in kidney tissue increased significantly at 3 h and peaked at 6 h after IR which were consistent with KLF6 mRNA expression (Figure 5(A–C)). Immunohistochemical staining showed that KLF6 was markedly up-regulated at tubular and mildly expressed in glomeruli at 6 h after IR versus the sham group, mainly located in the proximal and distal tubule (Figure 5(D)), suggesting that the overexpressed KLF6 is a potential biomarker for AKI.

### 3.4. Inflammation cytokines increased in IR model

**Figure 6.**
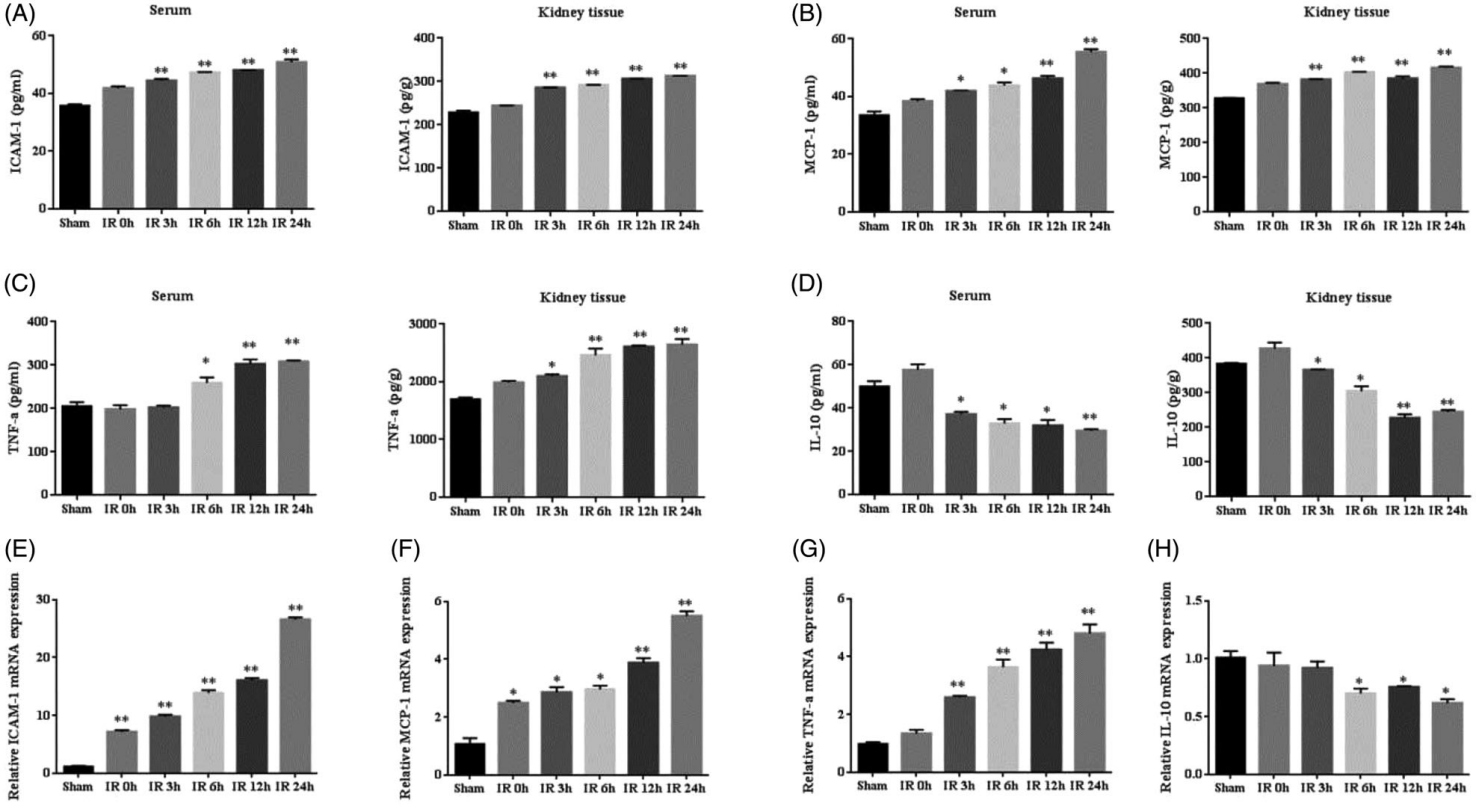
The expression of inflammation cytokines in IR model. (A–D) Quantitative analysis of ICAM-1, MCP-1, TNF-α, and IL-10 released in the serum and kidney tissue using ELISA. (E–H) Quantitative analysis of ICAM-1, MCP-1, TNF-α, and IL-10 mRNA expression in the kidneys by RT-PCR. *p < 0.05 versus sham; **p < 0.01 versus sham.

Studies have shown that inflammatory response is a major contributor to IR AKI. ELISA results indicated that renal IR induced sharp increases in the concentration of ICAM-1, MCP-1 and TNF-α and decrease in IL-10 in the serum and kidney tissue, reaching a peak at 24 h after IR (Figure 6(A–D)). Results of RT-PCR showed that renal IR led to a marked increase of ICAM-1, MCP-1 and TNF-α mRNA expression in the kidney tissue at 24 h, also reduced the mRNA expression of IL-10 (Figure 6(E–H)). These results indicate that IR initiate inflammatory response and the expression of pro-inflammatory cytokines increases and anti-inflammatory cytokine deceases.

### 3.5. Knockdown of KLF6 impairs the IR-induced inflammatory response in HK-2 cells

**Figure 7.**
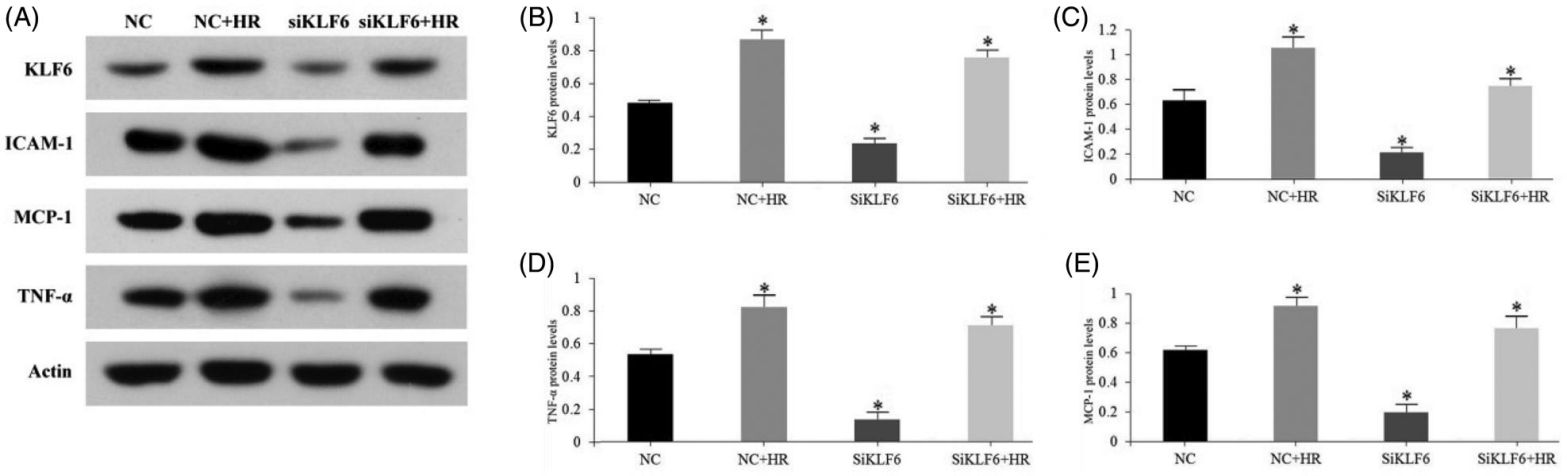
Knockdown of KLF6 expression reduced the HR-induced pro-inflammatory cytokines in HK-2 cells. (A–E) The expression of KLF6, ICAM-1, MCP-1, and TNF-α was measured by Western blot, in NC or siKLF6 cells with or without HR treatments. *p < 0.05 versus NC group; **p < 0.01 versus NC group.

**Figure 8.**
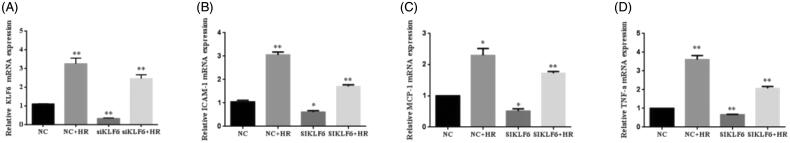
Knockdown of KLF6 expression reduced the HR-induced pro-inflammatory cytokines in HK-2 cells. (A–D) The expression of KLF6, ICAM-1, MCP-1, and TNF-α was measured by RT-PCR, in NC or siKLF6 cells with or without HR treatments. *p < 0.05 versus NC group; **p < 0.01 versus NC group.

To further study whether KLF6 is involved in kidney inflammation, we constructed an *in vitro* model of hypoxia-reoxygenation (HR) injury. RT-PCR and Western blot analysis showed that HR treatment significantly increased KLF6 expression in HK-2 cells, while the expression of KLF6 in HK-2 cells was significantly decreased after KLF6 siRNA transfection. Moreover, KLF6 knockdown inhibited ICAM-1, MCP-1 and TNF-α mRNA and protein levels response to HR injury (Figure 7  and Figure 8). These findings suggest that inhibition of KLF6 can reduce HR-induced pro-inflammatory cytokines secretion of HK-2 cells .

## 4. Discussion

AKI is a common syndrome characterized by a sudden drop in glomerular filtration rate, manifested by increased serum creatinine concentration or oliguria [34]. The main complications include volume overload, electrolyte imbalance, uremia, and so on. IR injury is a major cause of AKI and renal failure, causing high mortality worldwide [35]. During the early stage, most ischemic AKI is reversible, so further research is essential for early intervention to prevent and reduce AKI. At present, serum creatinine (Scr), urea nitrogen (BUN), and some protein indicators are used as a routine basis for the diagnosis of AKI. However, they all have a certain hysteresis and a lack of specificity, which are unable to identify the ultra-early stages of renal dysfunction. Therefore, ultra-early indicators are urgently needed for diagnosis of AKI to improve early prevention and treatment [36]. Our studies have shown that after renal IR injury, the level of Scr reaches the highest at 24 h, pathological damage is also the heaviest, accompanied by infiltration of inflammatory cells and activation of inflammatory cytokines (increased pro-inflammatory cytokines, decreased anti-inflammatory cytokines) [37]. However, bioinformatics analysis showed that KLF6 was involved in many critical pathways such as cell activation, leukocyte activation and response to hydrogen peroxide(H_2_O_2_) response to renal IR, while KLF6 was highly expressed at 3 h of IR than at 24 h. Our research proves that the mRNA and protein expression of KLF6 in renal tissues and serum respectively proved direct experimental data support, which demonstrates that the results of the gene analysis in this study is correct. The KLF6 increased gradually after IR and reached the highest level at 6 h, then began to decline, and earlier than the change of Scr. Our results also clearly showed that hypoxia induces increased KLF6 expression in HK-2 cell, which is consistent to the *in vivo* data. These results suggest that KLF6 may be a new marker of AKI and could be used for early diagnosis of AKI.

Ischemic AKI is the most common cause of AKI, and inflammatory responses are inevitably involved in. In the process of ischemic AKI, multiple factors are involved in activating and recruitment of immune cell to the injured kidney. These factors include danger-associated molecular patterns (DAMPs) and hypoxia-inducible factors (HIFs), increased expression of adhesion molecules, the production of chemokines and cytokines, activation of complement system and Toll-like receptors (TLRs) as well as the permeability dysfunction of the renal vascular endothelium. Immune cells of both the innate and adaptive immune systems, such as neutrophils, macrophages, and lymphocytes contribute to the pathogenesis of renal injury after IR, with some of their subpopulations also participating in the repair process [38].

Recent studies have provided evidence that TEC-associated inflammation aggravates kidney injury. First, TECs produced multiple inflammatory factors, triggered, and amplified inflammation. Second, TECs expresses TLRs, which critically contribute to activation of the complement system and recruitment of immune cells in response to inflammatory stimuli. For instance, TLR2 and TLR4 are expressed on normal renal TECs and their expression further increases after IRI. In addition, DAMPs such as histones or high-mobility-group protein B1 released from necrotic tubules can also activate TLRs on dendritic cells or macrophages and inflammasomes in the cytosol to trigger the secretion of proinflammatory cytokines and chemokines in the post-ischemia kidney [39–42]. Simultaneously, intra-renal activation of HIFs occurs in tubular, interstitial, and endothelial cells following IRI. Upregulation of HIF-1α occurs within 1 h and is sustained up to 7 days, and induces the infiltration of macrophages following IRI [43]. Through the above process, TECs are widely involved in inflammation. The cell culture results of our study showed that knockdown of KLF6 in HK-2 cells reduced the expression of ICAM-1, TNF-α, MCP-1, indicating that KLF6 may participate in the mechanism of renal IR by regulating the expression of inflammatory cytokines in TECs.

Most previous studies have demonstrated that large amounts of reactive oxygen species (ROS) produced in IR-induced AKI lead to TECs damage, thus, activating the inflammatory signaling pathway and triggering a cascade of inflammatory responses. H_2_O_2_, as one of ROS, has become an important tool for studying various types of cell oxidative damage recently. The bioinformatics analysis showed that KLF6 was involved in H_2_O_2_ response to renal IR. ICAM-1 that appears to be mediated by H_2_O_2_ response, which in response increases neutrophils rolling, adhesion, infiltration and its retention which induces an inflammatory response and, thus, increases the extent of injury [44]. TNF-α are regarded as important inflammatory mediators in the early stages of AKI, and have been confirmed to regulate the activity of helper T lymphocytes, the accumulation of neutrophils, macrophages, and lymphocytes, and as mediators of the inflammatory response. MCP-1 is a small chemokine, which recruits monocytes, memory T cells, and dendritic cells to the sites of inflammation produced by either tissue injury or infection [8]. The transcription factor NF-κB plays a pivotal role in abroad range of physiological and pathological processes, including development, inflammation, and immunity [26]. NF-κB plays an important role in the inflammatory response by regulating the expression of cytokines, adhesion molecules, and chemokines [45]. Zhang et al. found that KLF6 interacts with NF-κB p65 in the nucleus and binds to the promoters of p65 target genes, which is required for the optimal binding of p65 to the promoters and subsequent transcription of a subset of downstream genes, including MCP-1, CXCL2, and IL-8 [26]. Our study revealed that ischemic AKI involves the generation of cytokines and chemokine, including ICAM-1, TNF-α, MCP-1, and infiltration of the kidney by neutrophils and macrophages. The cell culture results in this study showed that knockdown of KLF6 reduced the expression of ICAM-1, TNF-α, MCP-1, indicating that KLF6 may participate in the mechanism of renal IR by regulating the expression of inflammatory cytokines in TECs and play a protective role on IR.

It is well characterized that macrophages act as key players in renal injury, inflammation, and fibrosis [46]. Animal models demonstrate that the macrophage is a major contributor to the inflammatory response to AKI. Emerging data from human biopsies also demonstrate the presence of macrophages in AKI and their persistence in progressive chronic kidney disease. Monocytes infiltrate the injured kidney shortly after neutrophils, differentiate into macrophages, and contribute to early tubular injury. During the early phase, DAMPs, as well as pro-inflammatory cytokines, which promote full activation of the pro-inflammatory M1 macrophage. Pro-inflammatory macrophages produce a large amount of TNF-α, ROS and other proinflammatory mediators that amplify inflammation and promote additional injury in a positive feedback loop. M1 macrophages may convert to M2 macrophages in response to tissue factors within the kidney during the recovery phase. Anti-inflammatory M2 macrophages also suppress kidney inflammation and injury *via* secretion of anti-inflammatory cytokines such as IL-10 and TGF-β [12, 47–49]. Our study demonstrated that the concentration of IL-10 decreased in the serum and kidney tissue, reaching a peak at 24 h after IR, which have converse changes with pro-inflammatory cytokines.

Kim et al. demonstrated that KLF6 promotes pro-inflammatory gene programming in macrophages, and KLF6 deficiency significantly attenuates macrophage recruitment to sites of inflammation [23]. KLF6 can also cooperates with NF-κB to promote pro-inflammatory gene expression while inhibiting STAT3 to restrain anti-inflammatory gene expression in macrophages [24]. Dipali provided the evidence that KLF6 promotes an M1 phenotype through cooperation with NF-κB. Furthermore, KLF6 was also found to inhibit the M2 targets by suppressing PPAR-γ expression [50]. Therefore, KLF6 plays an important role in macrophages function in many inflammatory-related diseases.

To our knowledge, this is the first study to address the expression of KLF6 in AKI and the underlying mechanism of KLF6 involving in inflammation in AKI. In view of the inflammation process of AKI is complicated signaling network and potentially extensive cross-talk with other signaling molecules, we still require to carry on further study to clarify which signal pathway does KLF6 participate in the inflammatory process and whether it has binding sites with some inflammatory cytokines directly, possible mechanism of effect on macrophages.

In conclusion, our results of gene enrichment analysis (GSEA) showed that KLF6 was involved in the activation of leukocyte by H2O2 response in AKI. The results of a series of experiments in cell biology and molecular biology indicate that KLF6 can be a new early biomarker to diagnose AKI and participate in the mechanism of renal IR by regulating the expression of inflammatory cytokines. Our data highlight KLF6 as a potential candidate for the development of new strategies for the treatment of AKI.
